# A unique case of appendiceal diverticulum presenting with positive faecal immunochemical test

**DOI:** 10.1093/jscr/rjae349

**Published:** 2024-05-30

**Authors:** Imad-ud-din Saqib, Nigel Noor, Heman Joshi

**Affiliations:** Department of General (Colorectal) Surgery, Cambridge University Hospitals NHS Foundation Trust, Addenbrooke’s Hospital, Hills Rd, Cambridge CB2 0QQ, United Kingdom; University of Cambridge, School of Clinical Medicine, Addenbrooke–s Hospital, Hills Rd, Cambridge CB2 0SP, United Kingdom; Department of General (Colorectal) Surgery, Cambridge University Hospitals NHS Foundation Trust, Addenbrooke’s Hospital, Hills Rd, Cambridge CB2 0QQ, United Kingdom; Department of General (Colorectal) Surgery, Cambridge University Hospitals NHS Foundation Trust, Addenbrooke’s Hospital, Hills Rd, Cambridge CB2 0QQ, United Kingdom

**Keywords:** appendiceal diverticulum, appendicitis, positive FIT, appendiceal mass, appendix

## Abstract

Appendiceal diverticulum is a rare condition that usually presents with symptoms similar to acute appendicitis. Although imaging can be used to aid the diagnosis of this condition, it is usually confirmed postoperatively on the basis of histology. Because of an increased risk of appendiceal neoplasms, the usual management is prophylactic appendicectomy. We report the case of a 70-year-old lady with no symptoms referred from her GP surgery for a positive faecal immunochemical test as part of the bowel screening programme. Colonoscopy showed a mass at the appendiceal orifice with normal histology. She underwent an appendicectomy with a small cuff of caecal resection. The lesion was ~8 cm at its maximum dimension and showed appendiceal diverticulum. Appendiceal diverticulum is an important differential diagnosis to consider in patients with atypical history of acute appendicitis or positive faecal immunochemical test with no other symptoms.

## Introduction

Appendiceal diverticulum is a rare condition that was first reported in the literature in 1893 [1]. Its frequency has been estimated to be ~0.04% to 3.7% [2–4]. The condition can be congenital or acquired depending on the layers involved [5].

The primary symptom is abdominal pain and the presentation is similar to acute appendicitis in majority of the cases [6]. The diagnosis is usually made postoperatively on histopathological analysis [7]. Appendiceal diverticulae are associated with a higher risk of perforation [2, 3] and appendiceal neoplasms [5, 8].

We report the case of an asymptomatic patient with a positive faecal immunochemical test (FIT) and a mass at appendiceal orifice on colonoscopy which showed normal mucosa and was diagnosed with an appendiceal diverticulum.

## Case report

A 70-year-old lady was referred by her General Practitioner (GP) surgery for a colonoscopy as part of the bowel screening programme for a positive FIT.

She had been participating regularly for the last 8 years. She had a single episode of bleeding per rectum 4 years back following which she had a FIT which was 15 μg/g. Her last normal FIT was 3 months back so the decision by her GP was to repeat another FIT in 2 months’ time which was normal.

She remained asymptomatic throughout this period with no history of abdominal pain, no change in bowel habits or irregular bowel habits, no anorexia or weight changes, and no bleeding per rectum. She had history of basal cell carcinoma of the skin which was resected. She had family history of bowel cancer in a first degree relative.

**Figure 1:**
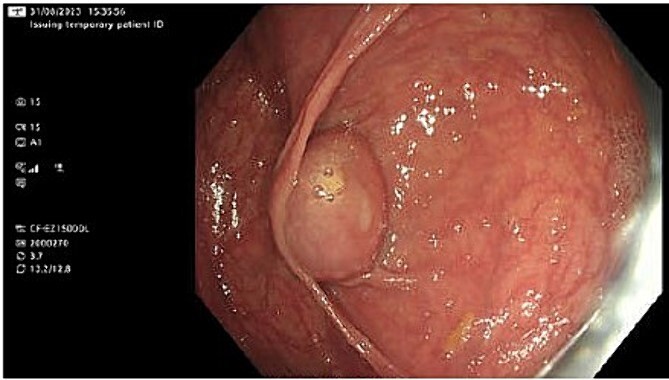
Mass at the appendiceal orifice similar to a mucocele.

She had a colonoscopy 2 weeks later. This showed a mass at the appendiceal orifice similar to a mucocele as shown in Fig. 1 from which biopsies were taken. Four polyps were also removed from the right colon, the largest being an 18 mm lesion in the ascending colon.

Histology showed normal colonic mucosa for the biopsies from the suspected mucocele, while the polyps were low grade tubular adenomas with one tubulovillous adenoma with low grade dysplasia.

**Figure 2:**
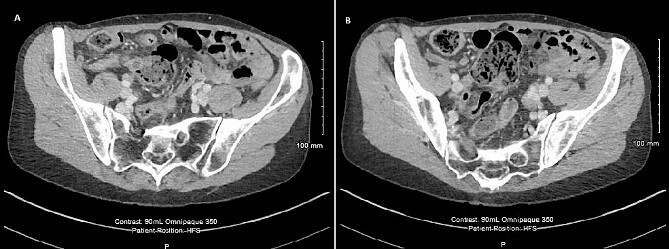
CT scan of the abdomen showing (A) 10 mm low attenuation lesion at the base of appendix and (B) mildly thick walled appendix containing a small volume of fluid.

Radiological imaging for the thorax, abdomen, and pelvis was done to further characterise the lesion at the appendix and any other associated lesions. This showed a 10 mm low attenuation lesion at the base of the appendix (Fig. 2A). The upstream appendix was normal in diameter at 6 mm but mildly thickened wall containing a small volume of fluid (Fig. 2B). A 2 mm left lower lobe nodule was also seen with appearances similar to an intrapulmonary node.

An incidental dilated ascending aorta and calcified aortic valve was also noted. An echocardiogram was done which showed dilated ascending aorta 4.5 cm with mild aortic regurgitation, mild mitral and tricuspid regurgitation, and a dilated right atrium.

The clinical details were discussed in the lower gastrointestinal multidisciplinary team meeting and planned for surgical resection. A laparoscopic appendicectomy plus a small cuff of caecal resection (31 mm × 11 mm) was done. There was a palpable ~6 mm lesion at the base of the appendix.

Macroscopically, the appendix measured 81 mm × 10 mm × 9 mm showing some dilatation of the lumen, with a small cuff of caecum as mentioned. The serosal surface appeared unremarkable. There was no evidence of exudate, mucin, or any site of perforation. On opening the caecal aspect, there was an obvious polypoid nodular intramural lesion within the appendix isolated to the base. Dissecting the appendix, there were thick cream secretions in the lumen but no definite mucin. The nodule measured 12 × 7 mm and on slicing contained cream soft material.

**Figure 3:**
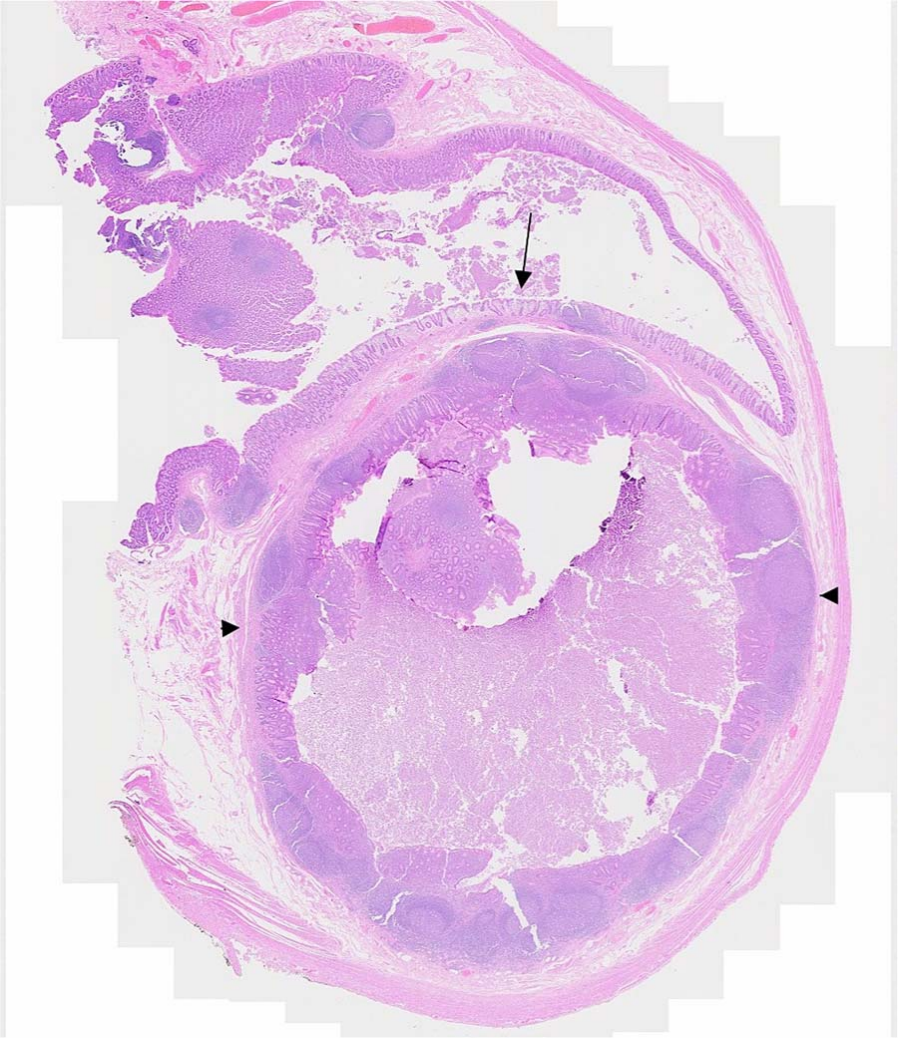
Microscopic section showing appendiceal diverticulum, Arrow: Caecal mucosa, Arrowheads: Submucosal lesion.

Microscopically (Fig. 3), sections of base of appendix and adjacent caecum showed a submucosal cystic lesion lined by large bowel mucosa with mucosal lymphoid follicle resembling appendiceal lining, in keeping with an appendiceal diverticulum at the base of appendix resembling a submucosal caecal lesion. Sections of the rest of appendix showed slight dilatation of the lumen with mildly inflamed mucosa, but were otherwise unremarkable. There was no evidence of dysplasia or malignancy.

The patient remained well postoperatively and discharged home on postoperative day 1 with no complications.

## Discussion

Appendiceal diverticulum is an uncommon entity that was initially described by Kelynack in 1893 [1]. The reported prevalence is considered to be ~0.04% to 3.7% [2–4] and identified in up to 2% of all appendicectomy specimens [6].

It can be either congenital or acquired depending on the number of layers herniating through the normal bowel wall. The congenital form of the disease, which is extremely rare, is considered to be similar to the diverticular disease of the colon. The exact mechanism of acquired disease, which is the predominant form, is not known. However, it is hypothesised to be secondary to obstruction of the orifice. Subsequent inflammation predisposes to the formation of false diverticula. As acquired diverticulae lack muscularis propria, the chances of perforation are approximately four times higher [6].

Potential risk factors include male gender, age greater than 30 years, chronic appendicitis, cystic fibrosis, and Hirschsprung’s disease. There is no known link between colonic diverticulae and appendiceal diverticulae [2, 9, 10].

Although symptoms vary, they usually mimic acute appendicitis presenting with central and right lower quadrant pain, pyrexia, nausea, emesis, anorexia, tenderness in the right lower quadrant and raised inflammatory markers in patients beyond their third decade of life. This usually occurs when there is acute appendiceal diverticulitis. In many instances, the abdominal pain is of a lower intensity, persists for a longer duration on further questioning, patients report having multiple previous episodes of pain of this nature [6]. In cases of perforation, they may be misdiagnosed as low grade appendiceal mucinous neoplasms as a result of serosal mucin deposits [7]. Only one case has been reported to present with haematochezia in the literature [11].

Although diagnosis is not as common preoperatively [7], ultrasonography, computed tomography scan, and barium enema study may be able to detect these diverticulae [9, 12, 13]. Ultrasonography findings include round cysts with enhanced walls attached to an enlarged appendix [12]. Histopathology postoperatively helps in establishing the final diagnosis.

Appendiceal diverticulae are classified into four types as shown in Table 1 [14].

**Table 1:** Types of appendiceal diverticulae.

**Type**	**Characteristics**
**Type 1**	Classic form, normal appendix, acutely inflamed appendiceal diverticulum
**Type 2**	Appendicitis with acutely inflamed appendiceal diverticulum
**Type 3**	Appendicitis with incidental non-inflamed appendiceal diverticulum
**Type 4**	Incidental non-inflamed appendiceal diverticulum with normal appendix

Management primarily involves appendicectomy [6, 14] in view of the increased risks of appendiceal neoplasms such as carcinoid tumours, mucinous adenomas, tubular adenomas, adenocarcinomas, and pseudomyxoma peritonei [5, 8]. However, the role of prophylactic appendicectomy has been questioned [9, 15].

Appendiceal diverticular disease is an important differential diagnosis to consider in patients with atypical history of acute appendicitis and incidental positive FIT in the absence of any other symptoms suggestive of a particular cause of bleeding.

## Conflict of interest statement

None declared.
